# Ion channel regulation by phosphoinositides analyzed with VSPs—PI(4,5)P_2_ affinity, phosphoinositide selectivity, and PI(4,5)P_2_ pool accessibility

**DOI:** 10.3389/fphar.2015.00127

**Published:** 2015-06-19

**Authors:** Alexandra Rjasanow, Michael G. Leitner, Veronika Thallmair, Christian R. Halaszovich, Dominik Oliver

**Affiliations:** ^1^Department of Neurophysiology, Institute of Physiology and Pathophysiology, Philipps UniversityMarburg, Germany; ^2^Institute of Physiology, University of FreiburgFreiburg, Germany

**Keywords:** Ci-VSP, lipid phosphatase, membrane-delimited signaling, microdomain, phosphoinositide, PI(4,5)P_2_, potassium channel

## Abstract

The activity of many proteins depends on the phosphoinositide (PI) content of the membrane. E.g., dynamic changes of the concentration of PI(4,5)P_2_ are cellular signals that regulate ion channels. The susceptibility of a channel to such dynamics depends on its affinity for PI(4,5)P_2_. Yet, measuring affinities for endogenous PIs has not been possible directly, but has relied largely on the response to soluble analogs, which may not quantitatively reflect binding to native lipids. Voltage-sensitive phosphatases (VSPs) turn over PI(4,5)P_2_ to PI(4)P when activated by depolarization. In combination with voltage-clamp electrophysiology VSPs are useful tools for rapid and reversible depletion of PI(4,5)P_2_. Because cellular PI(4,5)P_2_ is resynthesized rapidly, steady state PI(4,5)P_2_ changes with the degree of VSP activation and thus depends on membrane potential. Here we show that titration of endogenous PI(4,5)P_2_ with Ci-VSP allows for the quantification of relative PI(4,5)P_2_ affinities of ion channels. The sensitivity of inward rectifier and voltage-gated K^+^ channels to Ci-VSP allowed for comparison of PI(4,5)P_2_ affinities within and across channel subfamilies and detected changes of affinity in mutant channels. The results also reveal that VSPs are useful only for PI effectors with high binding specificity among PI isoforms, because PI(4,5)P_2_ depletion occurs at constant overall PI level. Thus, Kir6.2, a channel activated by PI(4,5)P_2_ and PI(4)P was insensitive to VSP. Surprisingly, despite comparable PI(4,5)P_2_ affinity as determined by Ci-VSP, the Kv7 and Kir channel families strongly differed in their sensitivity to receptor-mediated depletion of PI(4,5)P_2_. While Kv7 members were highly sensitive to activation of PLC by Gq-coupled receptors, Kir channels were insensitive even when PI(4,5)P_2_ affinity was lowered by mutation. We hypothesize that different channels may be associated with distinct pools of PI(4,5)P_2_ that differ in their accessibility to PLC and VSPs.

## Introduction

Phosphoinositides (PIs) are major determinants of subcellular membrane identity and directly control many aspects of cellular behavior, including membrane dynamics, dynamics of the cytoskeleton and electrical activity via control of ion channel activity (Di Paolo and De Camilli, 2006; Balla, 2013).

The ability to disrupt PI signals is pivotal for understanding the mechanistic role of these lipid signals and the complexities in the regulation of downstream targets. An obvious and widely used strategy has been to knock down or over-express the enzymes that generate, interconvert or consume the various PI isoforms. However, these approaches are usually effective on a slow timescale, i.e., many hours to days. Thus, it is often difficult to distinguish processes that directly depend on PIs from secondary cellular responses and compensatory mechanisms may mask relevant effects (Balla et al., 2009). Therefore, techniques for rapid intervention into Pi concentrations and their dynamics are required. Unfortunately, with the exception of PI-3-kinase inhibitors (Wymann and Schultz, 2012), potent and specific pharmacological tools that target PI kinases and phosphatases are mostly lacking (Balla, 2013). Several experimental approaches have been developed that allow for different degrees of spatiotemporal control of various PI species. One approach often used is the competitive binding of PIs by ligands that shield, and thus effectively eliminate PIs. These molecules include highly effective but unspecific polycationic chelators of PIs introduced into the cells, such as aminoglycosides or poly-lysine (e.g., Du et al., 2004; Oliver et al., 2004; Rapedius et al., 2007) and genetically encoded protein domains that bind to specific PIs with high selectivity (Arendt et al., 2009) but do not really allow for fast manipulation of PIs.

A versatile method for rapid alteration of PI concentration in living cells was independently introduced by two groups in 2006 (Suh et al., 2006; Szentpetery et al., 2010) and is now widely used. The essence of this approach is the overexpression of a PI-metabolizing enzyme, which is initially localized in the cytosol, thus having minimal impact on membrane PI levels. The enzyme can be recruited to the membrane by chemically inducing high-affinity hetero-dimerization of FKBP protein fused to the active enzyme and the FRB domain localized to the membrane via fusion to a short lipidated peptide as a “membrane anchor.” Recruitment by dimerization is induced rapidly by adding rapamycin or a rapamycin analog. Because both the enzyme effector construct and the membrane anchor are genetically encoded, they can be tailored to variety of needs in terms of the target membrane and the affected PI species (e.g., Szentpetery et al., 2010; Lindner et al., 2011; Hammond et al., 2012). On the one hand, the system is highly adaptable; on the other hand, however, it comes with an all-or-none mode of action. Once the dimerizer is added, the active construct is recruited to the membrane with high affinity, making the change in the PI concentrations practically irreversible and it gives little experimental control to set the degree of the changes in PI content. Recently, this system was combined with an optogenetic strategy, enabling localized and reversible recruitment induced by light, which potentially expands the range of questions that can be addressed experimentally (Idevall-Hagren et al., 2012).

An alternative strategy is to use voltage sensitive phosphatases (VSPs), which function as PI(4,5)P_2_ [and PI(3,4,5)P_3_] 5-phosphatases controlled by an N-terminal voltage sensor domain (Murata et al., 2005; Hossain et al., 2008). Upon depolarization they rapidly activate, enabling strong depletion of PI(4,5)P_2_ within a second or even less (Halaszovich et al., 2009; Falkenburger et al., 2010). Not surprisingly, VSPs were soon recognized as ideal tools to study the PI(4,5)P_2_ regulation of PI-effectors, especially of ion channels, since the electrophysiological voltage clamp approach used for control of the VSP easily accommodates simultaneous measurement of channel activity. Thus, many channels, ranging from Ca^2+^ channels (Suh et al., 2010), voltage gated (Falkenburger et al., 2010; Kruse et al., 2012), and two-pore domain K^+^ channels (Lindner et al., 2011) to TRP channels (Xie et al., 2011; Yudin et al., 2011) have been examined using the ascidian Ci-VSP or the homolog from zebrafish, Dr-VSP (Hossain et al., 2008). In contrast to the abovementioned methods, depletion of PI(4,5)P_2_ by VSPs is readily reversible, as VSP activity ceases upon repolarization to negative voltage, allowing endogenous PI-5-kinases to replenish the PI(4,5)P_2_ content of the membrane. Moreover, because steady-state concentration of PI(4,5)P_2_ at any given potential is determined by the counteracting activities of the VSP on the one hand and endogenous PI-5-kinase on the other hand (Falkenburger et al., 2010), it is possible to “titrate” the PI(4,5)P_2_ concentration by simply setting the membrane (Halaszovich et al., 2009).

Here we experimentally explore the possibility to use such VSP-mediated titration of PI(4,5)P_2_ to quantitatively estimate the apparent affinity of PI(4,5)P_2_ effectors for their lipid ligand. This affinity is biologically important, because it is considered as a major determinant of an effector's sensitivity to dynamic changes in cellular PI(4,5)P_2_ concentration (Du et al., 2004; Gamper and Shapiro, 2007; Hernandez et al., 2009). For example effectors with low affinity should be highly sensitive to the reduction in PI(4,5)P_2_ resulting from cellular signaling events.

Our results show that the apparent affinity of channels to endogenous PI(4,5)P_2_ can be precisely gauged by using VSPs for the titration of PI(4,5)P_2_. However, the findings also point out limitations of this approach that arise from non-specific PI interactions of some channel types. Moreover, we present preliminary evidence suggesting that different PI-metabolizing enzymes and PI(4,5)P_2_ effectors may have access to and interact with distinct populations or pools of PI(4,5)P_2_ within the membrane.

## Materials and methods

### Cell culture and molecular biology

Chinese hamster ovary (CHO) cells were cultured as described (Wissmann et al., 2003) and plated onto glass coverslips. Plasmids encoding Ci-VSP, channel subunits and the muscarinic receptor, M1R, were transfected into the cells with jetPEI transfection reagent (Polyplustransfection, Illkirch, France) according to the manufacturer's instructions. Briefly, transfection mixture containing a total of 3 μg plasmid DNA and 6 μl JetPEI transfection reagent was incubated at room temperature for 15–30 min and then added to a 35 mm dish. For co-transfection of constructs, equimolar ratios of DNA of each plasmid were used. The following expression plasmids were used: Ci-VSP was fused to CFP or RFP at its N-terminus (vectors pCFP-C1 or pRFP-C1, respectively) to facilitate identification of cells with robust expression and membrane localization. Channel subunits were cloned into pBK-CMV (Kir6.2, SUR1, Kir1.1) or pEGFP-C1 (Kir2.1, Kv7.2, Kv7.3). Mutations were generated using the QuikChange II XL Site-Directed mutagenesis kit (Stratagene, Agilent Technologies, Waldbronn, Germany). Plasmids encoding the rapamycin-sensitive recruitment system for Inp54p are described in Lindner et al. (2011) and PLCδ1-PH-GFP is described in Halaszovich et al. (2009). All constructs were verified by sequencing. Patch-clamp experiments were performed 24–48 h after transfection. For experiments with Ci-VSP, cells with clearly discernible membrane-localized fluorescence were selected by visual inspection (Olympus BX50WI upright microscope, LumPlan/Fl 60x water immersion objective, *NA* = 0.9).

### Electrophysiology

#### CHO cells

Cells were whole-cell voltage clamped with EPC-9 or EPC-10 amplifiers controlled by PatchMaster software (HEKA, Lambrecht, Germany). Voltage command protocols are described in the respective figure legend. Patch pipettes were pulled from quartz or borosilicate glass to an open pipette resistance of 2–3 MΩ when filled with intracellular solution containing (mM): KCl 135, MgCl_2_ 2.5, CaCl_2_ 0.24, EGTA 5, HEPES 5, Na_2_ ATP 3, Na_3_GTP 0.1, Spermine 0.01, pH 7.3 (with KOH). The extracellular solution contained (mM): NaCl 144, KCl 5.8, NaH_2_PO_4_ 0.7, Glucose 5.6, CaCl_2_ 1.3, MgCl_2_ 0.9, HEPES 10, pH 7.4 (with NaOH). Experiments were performed at room temperature (≈24°C). InSolutionTM Rapamycin dissolved in dimethyl sulfoxide stock solution was purchased from Merck. Oxotremorine-M (Tocris, Ellisville/Missouri, USA) was dissolved in extracellular solution.

#### Xenopus oocytes

*Xenopus laevis* oocytes were removed surgically from adult females, dissected manually, and stored in solution containing (mM) NaCl 82.5, KCl 2.5, NaH_2_PO_4_ 1, PVP 0.5 g/l, CaCl_2_ 1, MgCl_2_ 1, HEPES 5, penicillin/streptomycin 10,000 units/ml, pH 7.3 (with NaOH). Channel subunits were either subcloned into vector pGem-He (Kir2.1, SUR1,) or pBF (Ci-VSP, Kir1.1, Kir6.2). cRNA was synthesized using the mMESSAGE mMACHINE Kit (Ambion, Life Technologies). Approximately 50 nl of cRNAs containing solutions were injected. Oocytes were treated with 0.5 mg/ml collagenase type II (Sigma-Aldrich) and incubated in storage solution at 18°C for 1–3 days before use. The follicle layer was removed with a pair surgical forceps immediately before two-electrode voltage clamp recordings. Additionally, the vitellin layer was removed for inside-out patch-clamp experiments.

Two-electrode voltage clamp recordings were performed with a Turbo tec-10 cx npi amplifier (npi electronic GmbH, Tamm, Germany). Patchmaster software was used for data acquisition. Experiments were performed at room temperature (22–24°C). The extracellular solution contained (mM) NaCl 114.5, KCl 3, CaCl_2_ 1.8, MgCl_2_ 0.9, HEPES 10, pH 7.3 (with NaOH). Electrodes were filled with a solution containing 3 M KCl.

Patch-clamp recordings from giant inside-out patches were performed as described previously (Oliver et al., 2004) with an EPC-9 or EPC-10 patch-clamp amplifier controlled by Patchmaster acquisition software. Experiments were performed at room temperature (22–24°C). Electrodes were pulled from thick-wall borosilicate glass tubing (outer diameter, 2 mm; inner diameter, 1 mm) (glass: Hilgenberg, Malsfeld, Germany). Electrode tips were fire polished and briefly immersed in paraffin oil (Sigma-Aldrich, Germany), before back-filling with extracellular solution containing (mM) NaCl 117, KCl 3, CaCl_2_ 1, HEPES 10, pH 7.2 (with NaOH). Open pipette resistance was 0.2–0.5 MΩ. The cytoplasmic face of excised patches was superfused with either Mg^2+^-free intracellular solution containing (mM) KCl 100, EGTA 10, HEPES 10, pH 7.2 (with KOH) or intracellular solution containing (mM) KCl 116, MgCl_2_ 1.1, EGTA 2, HEPES 10, pH 7.2 (with KOH). Stock solutions (10 mM) of long-chain phosphoinositides (PI(4,5)P_2_ (Avanti Polar Lipids, Alabaster/Alabama, USA) and PI(4)P (Merck, Darmstadt, Germany) were prepared with intracellular solution without Mg^2+^ by sonication for 15 min twice and stored at −20°C. PI stock solutions were freshly dissolved each day into Mg^2+^-free intracellular solution to a final concentration of 10 μM and sonicated (2 × 10 min) before application to the cytoplasmic face of giant patches by a custom-made multi-barreled application system.

### TIRF imaging

TIRF imaging was done while whole-cell patch-clamping the cell under observation as described before (Halaszovich et al., 2009). Briefly, a BX51WI upright microscope (Olympus) equipped with a TIRF-condenser (NA 1.45; Olympus) and a 488 nm laser (20 mW; Picarro, Sunnyvale, California/USA) was used. Fluorescence was imaged through a LUMPlanFI/IR 40x/0.8 NA water-immersion objective. Images were acquired with a TILL-Imago QE cooled CCD camera (TILL photonics, Gräfelfing, Germany) in combination with a Polychrome IV light source (TILL photonics) controlled by TILLvision software (TILL photonics). The frame interval was 6 s and the laser shutter was controlled by the Polychrome IV. Experiments were carried out at room temperature (≈24°C). Imaging data were analyzed using TILLvision (Till photonics) and IgorPro (Wavemetrics). Regions of interest (ROIs) encompassed the footprint of a single cell excluding cell margins to avoid movement artifacts. F/F_0_-traces were calculated from the background-corrected TIRF signal intensity F, normalized pixelwise to the initial intensity F_0_, which was calculated as the average over the baseline interval, by averaging over the ROI.

### Data analysis and statistics

Data were analyzed using Patchmaster software and IgorPro (Wavemetrics, Lake, Oswego, Oregon/USA). Steady state deactivation curves obtained during Ci-VSP experiments were calculated after 1 min of depolarization and fitted with the Boltzmann function I = I_min_ + (I_max_ − I_min_)/(1 − exp((V − V_1/2_)/s)), where I denotes the current amplitude, V is membrane voltage, V_1/2_ is the voltage at half-maximal current inhibition and s is the slope of the curve. Data were normalized to the average of the baseline currents obtained before depolarization. The time constants (τ) were obtained from monoexponential fits. All data are given as ± standard error of the mean (SEM). Statistics were calculated with IBM SPSS 17.0 (IBM Corporation, Somers/New York, USA) for MS Excel. Gaussian distribution was verified for all data by the use of the Kolmogorow–Smirnow-test. Because all data were normally distributed, differences were tested with student's *t*-test. Asterisks represent significant differences between *p* < 0.05 and 0.001.

## Results

### Kir channels

**Figure 1:**
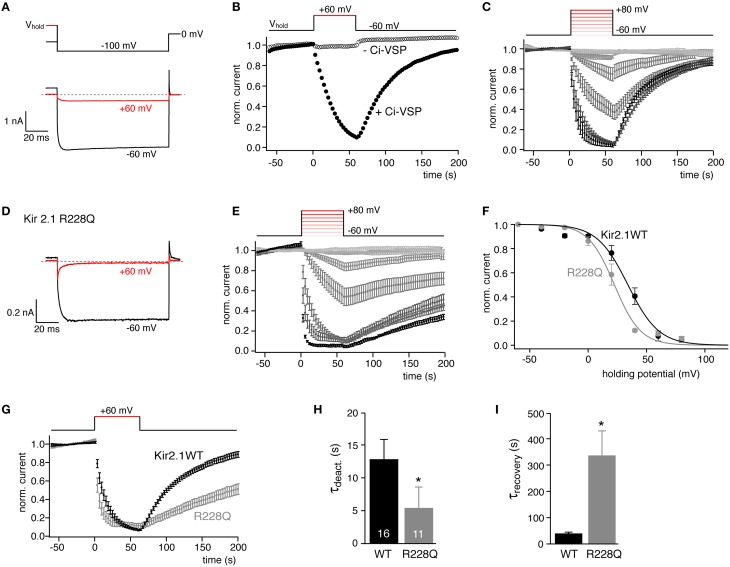
**Quantitative determination of the sensitivity of Kir2.1 to activation of Ci-VSP in CHO cells. (A)** Representative current recordings from CHO cells co-transfected with Kir2.1 channels and Ci-VSP (*black*). Holding potential was either −60 mV (black) or +60 mV (red) to activate Ci-VSP. Kir2.1 inward currents were evoked by briefly stepping to −100 mV as indicated in the schematic voltage protocol (upper panel). **(B)** Time course of K^+^ inward currents at −100 mV measured as in **(A)** when changing holding potential from −60 to +60 mV as indicated. Channel deactivsation was only observed when Ci-VSP was co-expressed (filled circles), but not in the absence of Ci-VSP (open circles). Currents were normalized to amplitude before switching the holding potential. **(C)** Current recordings obtained as in **(B)**. Holding potential was stepped to depolarized potentials between −40 and +80 mV (20 mV increments) as indicated (inset) Ci-VSP was activated at potentials from −60 to +80 mV with 20 mV increments. Data are mean ± SEM from *N* = 10–16 cells. **(D)** Representative currents recordings of cells co-expressing Kir2.1(R228Q) mutant channels and Ci-VSP with (+60 mV, *red*) and without (−60 mV, *black*) depolarization of the holding potential. **(E)** Normalized currents (mean ± SEM, *N* = 6–11) through Kir2.1(R228Q), recorded as in **(C)**. **(F)** Mean residual current amplitude at the end of each depolarizing period of holding potential is plotted as a function of Ci-VSP activation (holding) voltage yielded characteristic sigmoidal IV relations. Continuous lines indicate fits of a Boltzmann function to the data. **(G)** Comparison of time course of Kir2.1 and Kir2.1 R228Q current deactivation and recovery in response to potential-dependent activation Ci-VSP (data replotted from **C,E**). **(H,I)** Mean time constants (± SEM) for current deactivation and recovery in response to activation and deactivation of Ci-VSP by stepping the holding potential between −60 and +60 mV as in **(E)**. Time constants were derived from mono-exponential fits to individual recordings. Numbers of experiments (individual cells) as indicated. Asterisks indicate *p* ≤ 0.05.

We started our examination with Kir2.1, an inward rectifier K^+^ channel known to depend on PI(4,5)P_2_ for its activity (reviewed by Logothetis et al., 2007). Kir2.1 was previously shown to be down-regulated by activation of Ci-VSP (Murata and Okamura, 2007; Sakata et al., 2011). When Kir2.1 channels were co-expressed with Ci-VSP in CHO cells, strong depolarization resulted in deactivation of the current (Figures 1A,B). In contrast, when Kir2.1 was subjected to the same voltage protocol in the absence of Ci-VSP (Figure 1B) or when co-expressed with a catalytically dead mutant of Ci-VSP (data not shown), channel activity was essentially voltage independent. These experiments confirmed that channel deactivation resulted from depolarization-induced phosphatase activity of Ci-VSP, consistent with PI(4,5)P_2_ requirement for Kir2.1 activity. Following repolarization to −60 mV, currents recovered, reflecting PI(4,5)P_2_ resynthesis by endogenous PI-5-kinase (Halaszovich et al., 2009; Falkenburger et al., 2010).

We previously showed that Ci-VSP can be used to titrate plasma membrane PI(4,5)P_2_ content, such that increasing depolarization results in gradually decreasing PI(4,5)P_2_ concentration (Halaszovich et al., 2009). The underlying principle is that at any given membrane potential steady-state PI(4,5)P_2_ concentration is determined by the activity of endogenous (constitutively active) PI-5-kinase and the antagonistic 5-phosphatase activity of the VSP, the latter being directly voltage dependent (Falkenburger et al., 2010).

Here, we used such quasi-titration of endogenous PI(4,5)P_2_ to obtain a quantitative measure for the PI(4,5)P_2_ affinity of Kir2.1. Current was measured as a function of holding voltage in cells co-expressing Ci-VSP. As shown in Figure 1C, both the rate and degree of channel inhibition increased markedly with the level of depolarization. Plotting the current at the end of each depolarization against membrane potential yielded sigmoidal pseudo-dose-response curves (Figure 1F). A Boltzman function was empirically fitted to the data (see Materials and Methods) yielding the voltage that resulted in half-maximal inhibition (V_1/2_) as a measure for the sensitivity of the channels to depolarization and hence activation of Ci-VSP. Across many cells, V_1/2_ was highly reproducible (V_1/2_ = 34.1 ± 1.7 mV). We therefore considered V_1/2_ as a meaningful quantitative measure of the channel's sensitivity to depletion of PI(4,5)P_2_ and hence for the channel's apparent affinity for native cellular PI(4,5)P_2_.

To explore the general validity of this parameter as a proxy for phosphoinositide affinity, we aimed at comparing V_1/2_ between channels with different PI(4,5)P_2_ sensitivities. Various point mutations of Kir2.1 were shown to reduce the channel's apparent PI(4,5)P_2_ affinity as deduced from activation by the di-octanoyl PI(4,5)P_2_ analog, or from more indirect assays such as sensitivity to sequestration of PI(4,5)P_2_ by polycations or a PI(4,5)P_2_ antibody or sensitivity to receptor-induced activation of PLC (Lopes et al., 2002). Here we chose Kir2.1R228Q that according to the indirect assays displays a moderately reduced affinity for PI(4,5)P_2_ (Lopes et al., 2002) and may contribute to the phosphoinositide binding site of this channel (Logothetis et al., 2007). Kir2.1R228Q has indeed been used as a biosensor for Ci-VSP activity (Kohout et al., 2008, 2010). When subjecting Kir2.1R228Q to the same VSP activation protocol, the overall behavior was similar to the wild type channel (Figures 1D,E); however, inhibition occurred at more negative voltages, yielding a half-maximal inhibition at 21.6 ± 1.5 mV (Figure 1F), indicating that—consistent with a reduced affinity for PI(4,5)P_2_—deactivation of Kir2.1R228Q required less depletion of PI(4,5)P_2_ compared to wild type Kir2.1.

Therefore, V_1/2_ appears to be a useful measure for the PI(4,5)P_2_ affinity of ion channels and possibly other PI(4,5)P_2_ effectors.

In addition to the steady state channel activity, the kinetics of current deactivation and recovery also reflect the distinct PI(4,5)P_2_ affinities. As shown in Figures 1G–I, channel deactivation upon activation of Ci-VSP was faster in Kir2.1R228Q and channel reopening after switch-off of Ci-VSP was slowed nearly ten-fold. In culture cells such as CHO and HEK cells, recovery of the PI(4,5)P_2_ level after depletion by Ci-VSP requires some tens of seconds (Halaszovich et al., 2009; Falkenburger et al., 2010). Therefore, the slowed recovery probably also results from the reduced PI(4,5)P_2_ affinity rather than directly reflecting a slowed PI(4,5)P_2_ binding rate or channel kinetics.

**Figure 2:**
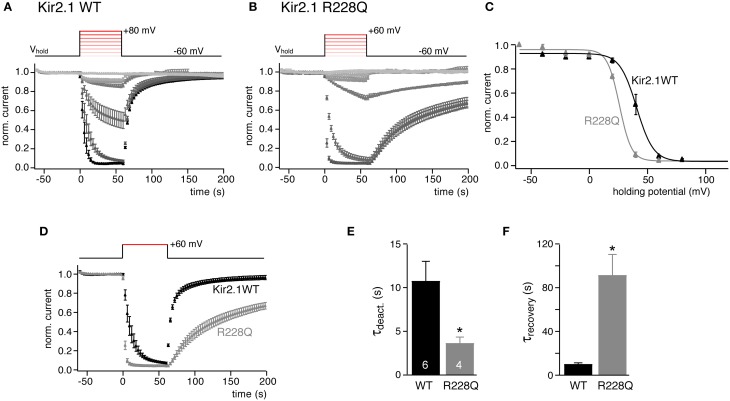
**Quantitative determination of the sensitivity of Kir2.1 to activation of Ci-VSP in** ***Xenopus*** **oocytes. (A)** Mean normalized K^+^ current amplitudes (±SEM) recorded from *Xenopus* oocytes co-injected with cRNAs encoding Kir2.1 and Ci-VSP. Oocytes were voltage clamped by two-electrode voltage clamp. Inward currents were measured during brief voltage jumps to 100 mV and changes in holding potential (inset) were applied as in Figure 1C. **(B)** Recordings as in **(A)** from X. oocytes co-expressing Kir2.1(R228Q) and Ci-VSP. **(C)** Plotting the mean residual current from the data shown in **(A,B)** reveals characteristic sigmoidal voltage-dependent inhibition of Kir2.1 and Kir2.1 R228Q currents. Continuous lines indicate fits of a Boltzmann function to the data. **(D)** Comparison of time course of Kir2.1 and Kir2.1 R228Q current deactivation and recovery in response to potential-dependent activation Ci-VSP (data replotted from **A,B**). **(E,F)** Mean time constants (±SEM) of current deactivation and recovery from the individual recordings shown in **(D)**. Time constants were derived from mono-exponential fits to individual recordings. Numbers of experiments (individual oocytes) as indicated. Asterisks indicate *p* ≤ 0.05.

We next tested if this method for estimating PI(4,5)P_2_ affinity can be generalized to be used with other cellular systems. Ci-VSP as well as ion channels have been studied extensively in the *Xenopus laevis* oocyte expression system. We therefore co-expressed Kir2.1 and Ci-VSP in oocytes and measured currents by two-electrode voltage-clamp. The same voltage protocols as in CHO cells were applied to quantify channel sensitivity to graded activation of co-expressed Ci-VSP. As shown in Figure 2, the results were essentially equivalent to the findings obtained in CHO cells. V_1/2_ was 40.7 ± 1.4 mV for Kir2.1 and shifted by 15 mV (to 25.9 ± 1.8 mV) for the Kir2.1R228Q mutant. Again, deactivation kinetics were accelerated nearly 3-fold and recovery time-course was slowed by about 10-fold in the mutant (Figures 2E,F). However, we note that while channel deactivation during Ci-VSP activation roughly matched the time course observed in CHO cells, recovery following repolarization was about 3-fold faster in oocytes both for wild type Kir2.1 and the Kir2.1R228Q mutant.

In conclusion, observations with Kir2.1 show that the voltage change required for channel inhibition and the kinetics upon changes in PI(4,5)P_2_ availability can be used to define a channel's PI(4,5)P_2_ affinity.

### KCNQ channels

We were interested in quantitatively comparing the sensitivity to Ci-VSP activity across different PI(4,5)P_2_ effectors. Therefore, we examined the response of Kv7 (KCNQ) channels, members of another well-studied class of PI(4,5)P_2_-sensitive channels. These voltage-gated K^+^ channels are different from inward rectifier channels in terms of their gating mechanisms and presumably their PI(4,5)P_2_ binding sites are structurally unrelated to the PI(4,5)P_2_ interaction sites of Kir channels (Hansen et al., 2011; Zhang et al., 2013). As with Kir channels, the sensitivity of Kv7 to Ci-VSP has been demonstrated previously, however without quantitatively comparing the response sensitivity between channel types and isoforms (Murata et al., 2005; Murata and Okamura, 2007; Villalba-Galea et al., 2009; Falkenburger et al., 2010; Lindner et al., 2011; Hobiger et al., 2012; Kruse et al., 2012).

**Figure 3:**
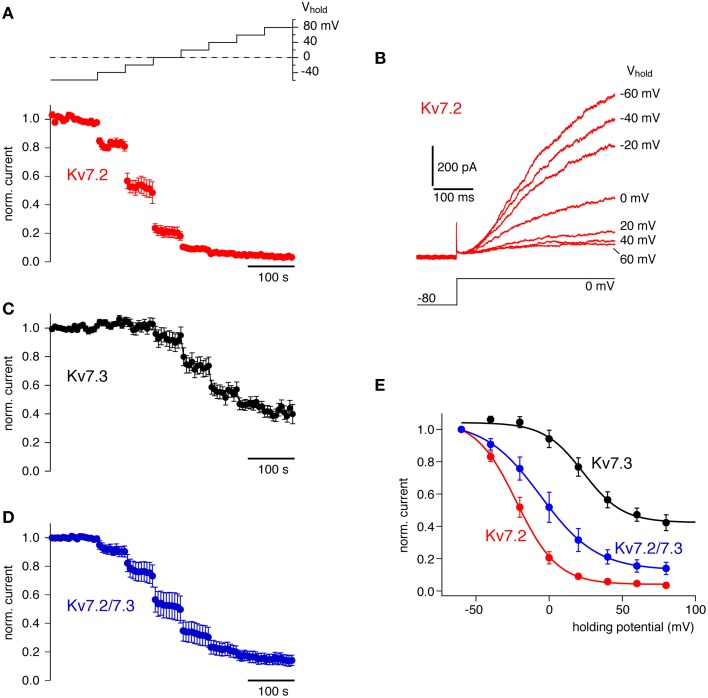
**Quantitative determination of the sensitivity of Kv7 channel isoforms to activation of Ci-VSP. (A)** Averaged whole-cell K^+^ currents from CHO cells expressing Kv7.2 together with Ci-VSP (mean ± SEM, *N* = 6 cells). Currents were probed at 6s-intervals with a voltage step to 0 mV preceded by brief hyperpolarization to deactivate channels. To activate Ci-VSP, the holding potential was varied between −60 and +80 mV in a stair-step manner (20 mV increments) as indicated by the inset. Currents are presented normalized to the amplitude at −60 mV holding potential before onset of the voltage stair. **(B)** Kv7.2 currents from a representative individual recording included in **(A)**. Currents shown were evoked by the voltage step protocol indicated at the end of each stair-step of the holding potential (as indicated for each trace). **(C)** Response of homomeric Kv7.3 channels to gradual activation of co-expressed Ci-VSP (*N* = 11 cells), recorded as in **(A)**. **(D)** Response of heteromeric Kv7.2/7.3 channels to gradual activation of co-expressed Ci-VSP (*N* = 8 cells), recorded as in **(A)**. **(E)** Mean residual currents at the end of each step of the holding-potential reveal the general sigmoidal dependence of current inhibition on the degree of activity of Ci-VSP and show distinct isoform-specific sensitivities. Continuous lines indicate fits of a Boltzmann function to the averaged data for each channel isoform.

We tested the response of homomeric Kv7.2 and Kv7.3, and heteromeric Kv7.2/7.3 channels to Ci-VSP-mediated depletion of PI(4,5)P_2_. As shown in Figure 3, depolarization of cells co-expressing Kv7 channels with Ci-VSP resulted in a gradual current decrease. All channels shared a sigmoidal dependence of activity on membrane potential that resembled the response of Kir2.1 channels to Ci-VSP activity. The individual channel isoforms displayed distinct sensitivities to Ci-VSP activation. Thus, Kv7.2 deactivated at weaker depolarization (V_1/2_ = −21.8 ± 3.9 mV) whereas Kv7.3 required stronger depolarization for current inhibition (V_1/2_ = 23.3 ± 2.7 mV). Moreover, a substantial fraction of the Kv7.3 current appeared insensitive to Ci-VSP-mediated depletion of PI(4,5)P_2_, at least within the accessible voltage range. Heteromeric Kv7.2/7.3 channels showed an intermediate sensitivity (V_1/2_ = −3.1 ± 7.5 mV). This pattern of responsiveness to Ci-VSP indicated a rank order of apparent PI(4,5)P_2_ affinities of Kv7.3 > Kv7.2/7.3 > Kv7.2. This order is consistent with previous estimates of PI(4,5)P_2_ sensitivities based on application of soluble PI(4,5)P_2_ analog (Li et al., 2005; Hernandez et al., 2009), confirming titration of endogenous PI(4,5)P_2_ with Ci-VSP as a useful method to estimate the channel's affinity for this lipid.

### PI specificity: Kir6.2 as an example

**Figure 4:**
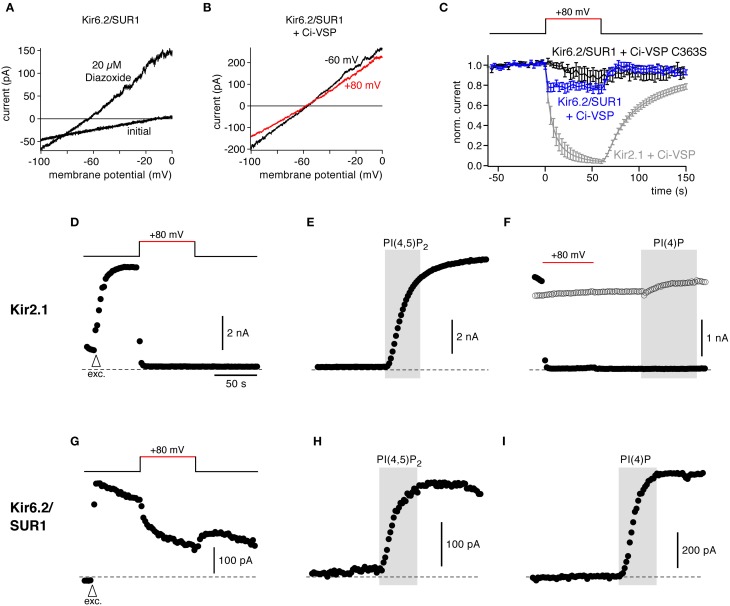
**Response to Ci-VSP is determined by PI selectivity, as revealed by the insensitivity of Kir6.2/SUR1 channels. (A)** Representative current recordings from a CHO cell expressing Kir6.2/SUR1. Channels were activated with 20 μM diazoxide in the presence of intracellular ATP, and currents were recorded in response to voltage ramps from −100 to 0 mV. **(B)** Kir6.2/SUR1 currents (with diazoxide) from a cell co-transfected with Ci-VSP without (black, holding potential −60 mV) and during (red, +80 mV) depolarization-dependent activation of Ci-VSP. **(C)** Time course of Kir6.2/SUR1 currents during depolarization of the holding potential recorded as in **(B)** in cells co-expressing either wild type Ci-VSP (blue) or catalytically inactive Ci-VSP (C363S) (black). Shown are mean outward currents at 0 mV to minimize influence of any leak currents, normalized to current amplitude before depolarization (± SEM, *N* = 12 and 10 cells, respectively). Activation of Ci-VSP had little effect on Kir6.2/SUR1 channels. Strong inhibition of Kir2.1 currents under the same experimental conditions is shown for comparison (replotted from Figure 1C). **(D)** In giant inside-out patches excised from *Xenopus* oocytes, activation of Ci-VSP caused fast, complete, and irreversible deactivation of Kir2.1. Currents were measured in response to brief voltage ramps (−100 to −60 mV) repeated every 2 s, while holding potential was changed as indicated by the inset. To facilitate comparison, absolute currents rather than inward current are shown. Arrowhead (labeled “exc.”) indicates time of excision of the patch from the oocyte. **(E)** Application of PI(4,5)P_2_ (10 μM) to excised patches readily activated Kir2.1 channels after Ci-VSP-induced channel deactivation. **(F)** Application of PI(4)P to the patch neither activated (filled circles; after Ci-VSP activation) nor inhibited (open circles; without Ci-VSP activation) Kir2.1 currents. **(G)** Representative recording of Kir6.2/SUR1 currents from a giant inside-out patch excised from an oocyte co-expressing Ci-VSP. Voltage protocol as in **(D,E)**. **(H,I)** Application of either PI(4,5)P_2_ or PI(4)P readily activated Kir6.2/SUR1, following channel rundown in a solution containing 1 mM Mg^2+^.

Kir6 is another inward rectifier K^+^ channel that together with SUR subunits forms K_ATP_ channels. Kir6.2/SUR1 strongly depends on PI(4,5)P_2_ for channel activity and for setting its sensitivity to intracellular ATP (Hilgemann and Ball, 1996; Fan and Makielski, 1997; Baukrowitz et al., 1998; Shyng and Nichols, 1998). Activation of Ci-VSP, however, had little effect on Kir6.2/SUR1 (K_ATP_) currents in CHO cells under conditions where Ci-VSP readily inhibited Kir2.1 (Figures 4A–C).

This resistance of Kir6.2 activity to PI(4,5)P_2_ depletion appeared surprising, since previous findings, including the application of exogenous PI(4,5)P_2_ and PI(4,5)P_2_-chelating compounds, consistently suggested that the PI(4,5)P_2_ affinity of K_ATP_/Kir6 channels is substantially lower than that of Kir2.1 (Du et al., 2004; Tucker and Baukrowitz, 2008). However, at the same time Kir6 channels show a lower specificity for exogenous phosphoinositides. Besides PI(4,5)P_2_, other PI isoforms can activate Kir (Fan and Makielski, 1997; Rohacs et al., 2003). Specifically, exogenous PI(4)P activated K_ATP_, although with somewhat lower efficacy than PI(4,5)P_2_ (Fan and Makielski, 1997; Baukrowitz et al., 1998). It is important to point out that Ci-VSP does not simply remove PI(4,5)P_2_ from the membrane but as a PI-5-phosphatase rather stoichiometrically converts PI(4,5)P_2_ into PI(4)P (Halaszovich et al., 2009). Given that the concentrations of the other two PIs affected by Ci-VSP, i.e., PI(3,4,5)P_3_ and PI(3,4)P_2_, are much less abundant in the plasma membrane than PI(4)P and PI(4,5)P_2_ (Balla, 2013), the overall concentration of phosphoinositides in the plasma membrane remains essentially constant when Ci-VSP is activated. Hence, the lack of effect of Ci-VSP on Kir6 may be readily explained by equal or similar affinities for PI(4)P and PI(4,5)P_2_, i.e., by lack of specificity for PI(4,5)P_2_.

To scrutinize this conclusion, we performed additional measurements in giant inside-out patches excised from *Xenopus* oocytes. In this recording configuration, Ci-VSP is expected to be highly efficient in irreversibly depleting PI(4,5)P_2_ because of lack of PI(4,5)P_2_ resynthesis in the absence of ATP at the exposed cytoplasmic face of the membrane (Hilgemann and Ball, 1996). At the same time, the inside-out patch allows for exogenous replenishment of specific PI isoforms.

Figure 4D shows that in giant patches expressing Ci-VSP, Kir2.1 currents were rapidly and completely inhibited by depolarization, indicating activity of expressed Ci-VSP. Channel deactivation was faster than in the whole-cell recordings and loss of channel activity was irreversible, consistent with the lack of PI(4,5)P_2_ replenishment. In contrast, Kir6.2 currents showed only a small decrease during depolarization. The current recovered rapidly after return to negative holding potential, indicating that this minor inhibition was not due to Ci-VSP-mediated depletion of PI(4,5)P_2_ (Figure 4G).

We next compared the differential response of both channels to Ci-VSP activity with their responsiveness to PI isoforms in the same experimental setting. To this end we applied normal (i.e., long-chain) PI(4,5)P_2_ and PI(4)P to the exposed intracellular face of the patches.

As shown in Figure 4E, PI(4,5)P_2_ strongly activated Kir2.1 after channel deactivation by Ci-VSP, similar to channel activation by PI(4,5)P_2_ following channel rundown due to phosphoinositide removal by endogenous enzymes. PI(4)P was entirely ineffective in activating Kir2.1 (Figure 4F), confirming the PI selectivity of this channel. It is worth noting that adding PI(4)P to channels without activating Ci-VSP did not inhibit channels (Figure 4F, open symbols), confirming that the effect of Ci-VSP on Kir2.1 results indeed from removal of PI(4,5)P_2_, not from accumulation of PI(4)P. In contrast to Kir2.1, both PI(4)P and PI(4,5)P_2_ similarly activated Kir6.2/SUR1 currents following rundown of endogenous phosphoinositides (Figures 4H,I).

In conclusion, these findings demonstrate that the responsiveness of a PI effector to Ci-VSP not only reflects its PI(4,5)P_2_ sensitivity (affinity) but also strongly depends on the isoform-specificity of the protein-lipid interaction.

### Can sensitivity to VSP predict the response to receptor-dependent PLC-β activity?

A physiologically important scenario of cellular PI concentration dynamics is the activation of PLC-β by a multitude of Gq/11-coupled receptors (Stauffer et al., 1998; Horowitz et al., 2005; Hernandez et al., 2009; Falkenburger et al., 2010). It is thought that this signaling pathway may act through depletion of PI(4,5)P_2_ by PLC-β. The degree of PI(4,5)P_2_ depletion that results from activation of Gq-coupled receptors may vary strongly dependent on factors such as receptor type, density and localization, the strength of PLC activation, and the rate of PI(4,5)P_2_ resynthesis. However, the sensitivity of the PI(4,5)P_2_ effector (ion channel) to a given degree of PI(4,5)P_2_ reduction should be mainly dependent on its affinity for PI(4,5)P_2_. Indeed, a correlation between the differential inhibition by receptors and the apparent affinity to PI(4,5)P_2_ has been demonstrated within specific classes of ion channels (Du et al., 2004; Hernandez et al., 2009) such that lower PI(4,5)P_2_ affinity results in higher sensitivity to activation of the GqPCR.

Therefore, we reasoned that the sensitivity to Ci-VSP should be a valid predictor of the responsiveness of PI(4,5)P_2_ effectors, e.g., ion channels, to physiological phosphoinositide signaling such as the activity of Gq-coupled receptors. To scrutinize this prediction, we tested the sensitivity of the channels examined above to activation of the prototypical Gq-coupled M1 receptor (M1R), a muscarinic acetylcholine receptor that robustly depletes PI(4,5)P_2_ when overexpressed in CHO cells (e.g., Hernandez et al., 2009; Lindner et al., 2011; Wilke et al., 2014). Muscarinic receptors are prominent regulators of endogenous K^+^ channels including Kv7 in a wide variety of neurons (e.g., Brown and Adams, 1980; Marrion et al., 1989; Suh and Hille, 2002; Broicher et al., 2008).

**Figure 5:**
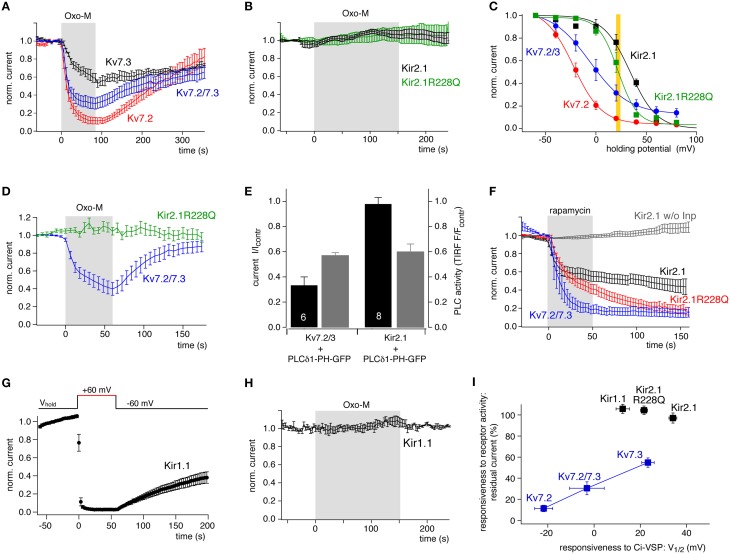
**Comparison of channel sensitivity to VSP and to PLC activity. (A)** Kv7 isoforms were inhibited robustly but differentially by stimulation of co-expressed M1R with Oxotremorine-M (10 μM). Whole-cell recordings from CHO cells were measured by voltage pulses from −60 to 0 mV every 6 s. Mean currents (±SEM) are presented normalized to amplitude before application of Oxo-M (*N* = 6, 5, 7 for Kv7.2, Kv7.3, and Kv7.3/7.3, respectively). **(B)** Kir2.1 or Kir2.1 R228Q channels coexpressed with M1R were not affected even by prolonged activation of M1R (mean ± SEM; *N* = 5 and 7, respectively). **(C)** Quantitative comparison of sensitivity of Kv7 and Kir2.1 channels to PI(4,5)P_2_ depletion by co-expressed Ci-VSP. Data replotted from Figures 1F, 3E. The yellow bar indicates the Ci-VSP activity level equivalent to the degree of M1R-induced Kv7 inhibition. This VSP activity corresponds to a degree of PI(4,5)P_2_ depletion sufficient for substantial inhibition of wild-type and mutant Kir2.1. **(D)** Kir2.1R228Q and Kv7.2/3 co-expressed in the same cells were differentially sensitive to activation of co-expressed M1R. Voltage protocol for isolating Kir and Kv currents consisted of a hyperpolarization to −120 mV at which inward Kir2.1 currents were measured, followed by a step to 0 mV, where outward Kv7 currents were activated but Kir channels closed (mean ± SEM; *N* = 9 cells). **(E)** Kv7.2/3 or Kir2.1 channels were co-expressed with the PLC activity sensor PLCδ 1-PH-GFP and M1R. Current levels (black) and membrane localization of PLCδ 1-PH (gray) were recorded simultaneously by whole-cell patch clamp and TIRF microscopy, respectively. Current or fluorescence amplitudes at the end of a 120 s application of Oxo-M are shown normalized to baseline signals immediately before activation of M1R (control). Note that the degree of PLC activity as reported by the reduction in the TIRF fluorescence is equal in experiments with both channels. Shown are mean ± SEM, numbers of independent experiments (cells) as indicated by numbers in the black bars. **(F)** Chemical recruitment of a specific PI(4,5)P_2_ 5-phosphatase (Inp54p) by application of 5 μM rapamycin (5 μM) induces inhibition of Kir2.1, Kir2.1(R228Q), and Kv7.2/7.3 channels in CHO cells (mean ± SEM, *N* = 9, 12, and 5, respectively). Lack of inhibition of Kir2.1 in cells without co-expression of the phosphatase construct (w/o Inp; gray; *N* = 7), demonstrates that inhibition results from depletion of PI(4,5)P_2_ but not from direct inhibition of the channel by rapamycin. **(G)** Kir1.1 channels expressed in CHO were strongly inhibited by co-expressed Ci-VSP. Voltage protocol as in Figure 1 (mean ± SEM; *N* = 11 cells). **(H)** Kir1.1 channels were insensitive to stimulation of co-expressed M1R in CHO cells (mean ± SEM; *N* = 4 cells). **(I)** Sensitivity of the various channels to PLC/receptor-mediated depletion of PI(4,5)P_2_ plotted vs. the sensitivity to activation of Ci-VSP (V_1/2_). A correlation is observed only for Kv7 channel isoforms. V_1/2_ (Ci-VSP) for Kir1.1 channels was obtained as shown for Kir2.1 in Figure 1.

As shown in Figure 5A, all Kv7 channel subtypes tested were robustly inhibited by activation of co-expressed M1R with a saturating concentration of the agonist, oxotremorine-M (Oxo-M, 10 μM). The degree of channel closure differed between Kv7 subtypes: Kv7.2 was inhibited most strongly whereas Kv7.3 was inhibited only by about 50% and Kv7.2/7.3 heteromeric channels showed intermediate behavior. The degree of inhibition was similar to that obtained previously in the same cell type (Hernandez et al., 2009). Notably, the receptor-dependent inhibition correlated closely with the PI(4,5)P_2_ sensitivity as deduced from Ci-VSP response. This is nicely seen when channel inhibition is plotted as a function of V_1/2_ obtained from Ci-VSP experiments (Figure 5I), thus supporting the idea that sensitivity to Ci-VSP is a meaningful predictor of physiological behavior of the PI(4,5)P_2_ effector.

We next tested the sensitivity of Kir2.1 channels to muscarinic activation of PLC (Figure 5B). In contrast to Kv7 channels, Kir2.1 was entirely insensitive to stimulation. This may be explained by its higher affinity for PI(4,5)P_2_ reflected by the more positive V_1/2_ of Ci-VSP response, which is consistent with earlier estimates of relative PI(4,5)P_2_ affinity (Lopes et al., 2002; Du et al., 2004). The degree of depletion of PI(4,5)P_2_ obtained by M1R activation may enforce deactivation of Kv7 channels, but may still be permissive for full activity of Kir2.1. Yet, the complete insensitivity to PLC activation seemed surprising, since the V_1/2_ obtained with Ci-VSP was not dramatically more positive than for Kv7.3. As shown in an overlay of their Ci-VSP response curves (Figure 5C), PI(4,5)P_2_ depletion sufficient for suppression of Kv7.2/3 heteromeric channels to about 30% is expected to lead to a detectable deactivation of Kir2.1 as well.

We therefore tested Kir2.1R228Q for its sensitivity to receptor-induced PI(4,5)P_2_ depletion (Figure 5B). Surprisingly, despite its lowered PI(4,5)P_2_ affinity, Kir2.1R228Q was also entirely insensitive to activation of M1R. According to its response to Ci-VSP, the PI(4,5)P_2_ affinity of the Kir2.1 mutant closely matches that of Kv7.3, which is reliably inhibited by M1R activation. To exclude that the differential response of the channels result from differences in PLC expression or activity depending on the over-expressed channel type, we examined the muscarinic response of Kv7.2/3 and Kir2.1R228Q channels co-expressed in the same cells (Figure 5D). Again, Kv7 currents were strongly suppressed by M1R activation, whereas synchronously monitored Kir2.1R228Q currents were entirely unaffected. The same result was obtained when wild-type Kir2.1 was coexpressed with homomeric Kv7.2 (not shown). As an additional control, we quantified the PLC activity induced by M1R activity in cells expressing either Kv7 or Kir channels. To this end, the channels were individually co-expressed with the PI(4,5)P_2_/PLC sensor PLCδ 1-PH-GFP. This sensor binds to PI(4,5)P_2_ and is therefore localized at the plasma membrane in the resting cell, but dissociates into the cytosol upon activation of PLC, quantitatively indicating the degree of PI(4,5)P_2_ hydrolysis (Varnai and Balla, 2006). Membrane association of the PLCδ 1-PH-GFP was quantified by TIRF microscopy (Halaszovich et al., 2009), such that a reduction in the fluorescence signal indicated translocation into the cytosol and hence PLC activity and depletion of PI(4,5)P_2_. Fluorescence signal and channel activity were monitored simultaneously by TIRF and whole-cell voltage-clamp, respectively. As summarized in Figure 5E, cells expressing either Kv7.2/3 or Kir2.1, exhibited the same degree of PLC-mediated PI(4,5)P_2_ cleavage, whereas only Kv7.2/3 channels were suppressed by M1R activity. Therefore, differences in PLC activity can be excluded as a source of the differential response of both channel types.

Although 3-phosphorylated PIs are only a minor fraction of the overall PI content of the plasma membrane (Balla, 2013), one concern was that the sensitivity of Kir channels to Ci-VSP may also be influenced by Ci-VSP's enzymatic action on PI(3,4,5)P_3_ (Halaszovich et al., 2009). Therefore, we performed an additional set of control experiments, replacing Ci-VSP by a chemically recruitable PI(4,5)P_2_ phosphatase (Suh et al., 2006; Varnai et al., 2006; Lindner et al., 2011). Although in this approach, PI(4,5)P_2_ cannot be controlled in a graded manner and depletion is irreversible, the recruited Inp54p phosphatase selectively converts PI(4,5)P_2_ to PI(4)P without interfering with other PIs. As shown in Figure 5F, not only Kv7.2/7.3 channels but also Kir2.1 was robustly and invariantly inhibited by this maneuver. The inhibition developed more slowly and the degree of inhibition was slightly less complete for Kir2.1 channels, which is consistent with its higher affinity for PI(4,5)P_2_. The findings also recapitulate the effects observed after activation of pseudojanin (Kruse et al., 2012), a PI phosphatase that depletes both, PI(4,5)P_2_ and PI(4)P (Lindner et al., 2011). Inhibition of Kir2.1R228Q was similar to the effect of Inp54p recruitment on Kv7.2/7.3. Therefore, depletion of PI(4,5)P_2_ alone is indeed sufficient for inhibiting Kir2.1 channels.

Another Kir channel isoform, Kir1.1 (ROMK) responded readily to Ci-VSP activation (Figure 5G). The V_1/2_ of Ci-VSP inhibition was 12.4 ± 2.9 mV, placing the apparent affinity of Kir1.1 well in the range of Kv7 channels (between Kv7.3 and the more sensitive Kv7.2/3 heteromers). However, as seen for Kir2.1 channels, Kir1.1 currents in CHO cells were unaffected by activation of co-expressed M1R (Figure 5H).

Figure 5I summarizes these findings: despite similar sensitivity of Kir and Kv7 channels to PI(4,5)P_2_ depletion by the 5-phosphatases VSP and Inp54p, only Kv7 channels deactivate when PLC-β depletes PI(4,5)P_2_. In contrast, Kir channels are insensitive to receptor/PLC induced PI(4,5)P_2_ dynamics, and do not follow the same correspondence between V_1/2_ and inhibition by the receptor established for Kv7 channels. We conclude that other factors beyond PI(4,5)P_2_ affinity may contribute to the response sensitivity of these PI(4,5)P_2_ effectors to Gq/PLC signaling. We hypothesize that Kir2.1 is associated with a PI(4,5)P_2_ pool inaccessible to PLC-β, whereas Kv7 channels require a PI(4,5)P_2_ pool for their activity that is sensitive to PLC-β activity.

## Discussion

### VSPs to quantify PI(4,5)P_2_ affinity

Since their discovery (Murata et al., 2005), VSPs have become increasingly popular tools to study PI(4,5)P_2_-dependent processes. So far, VSPs have mostly been used to define the dependence of ion channels on this membrane lipid. Because an electrophysiological voltage clamp approach is used for control of the VSP, simultaneous measurement of ion channel activity is also straightforward. Moreover, usage of Ci-VSP to analyze other cellular processes seems also feasible (Yamaguchi et al., 2014). The depolarization of the membrane required for activation of a VSP may be achieved extracellular K^+^ concentration or activation of depolarizing ion channels by application of their ligands (Mavrantoni et al., 2015).

However, so far VSPs have mostly been used in a qualitative manner, i.e., to demonstrate the dependence or independence of channel activity on PI(4,5)P_2_, rather than quantitatively characterizing the protein's sensitivity to the lipid concentration. A more quantitative approach based on the time course of channel inhibition has been used by the Hille group (Falkenburger et al., 2010). It is based on the idea that following activation of Ci-VSP by a voltage step the PI(4,5)P_2_ concentration drops rapidly but gradually and it recovers after repolarization more slowly as PI-5-kinases replenish PI(4,5)P_2_ (Halaszovich et al., 2009). Thus, a low-affinity channel is expected to deactivate rapidly and to recover slowly. Consistent with this idea, we showed here that among Kir2.1 channel variants the high affinity WT channel exhibits slower deactivation and faster reactivation upon activation and deactivation of Ci-VSP, respectively (Figure 1G). However, an important caveat should be pointed out: the kinetics are a meaningful proxy for the effector's PI(4,5)P_2_ affinity only if binding equilibration with membrane PI(4,5)P_2_ and gating kinetics of the channel are much faster than the changes in PI(4,5)P_2_ induced by the VSP (Suh et al., 2010). While this may be case for many ion channels, the cellular effects of many PI(4,5)P_2_-binding proteins may be much slower.

In contrast, here we use variation of the membrane potential to control the activity of Ci-VSP in a graded manner (Murata and Okamura, 2007; Sakata et al., 2011). Because in the presence of (overexpressed) VSP, PI(4,5)P_2_ concentration is set by the opposing activities of the VSP at any particular voltage (plus endogenous voltage-independent PI-5-phosphatases) on the one hand and endogenous voltage-independent PI-5-kinase on the other hand, it is possible to gradually set or “titrate” the steady-state concentration of PI(4,5)P_2_ by changing membrane potential (Halaszovich et al., 2009; Falkenburger et al., 2010).

As shown here, this approach allows for a reproducible, quantitative assessment of the apparent PI(4,5)P_2_ affinity of ion channels and allows for comparison of apparent affinities across different classes of PI(4,5)P_2_ effectors. Previously, differential sensitivity to graded activation of VSPs was also observed for various TRPC channel isoforms (Imai et al., 2012) and for the PI(4,5)P_2_ sensors tubby-GFP and PLCδ 1-PH-GFP (Halaszovich et al., 2009). It should be possible to adapt this approach for most other PI(4,5)P_2_ effectors irrespective of the PI(4,5)P_2_ effector's kinetics. Ion channels that inactivate in a voltage-dependent manner may be an exception, as complete inactivation may prevent observation of channels activity during the depolarizing protocols required to activate VSPs.

**Figure 6:**
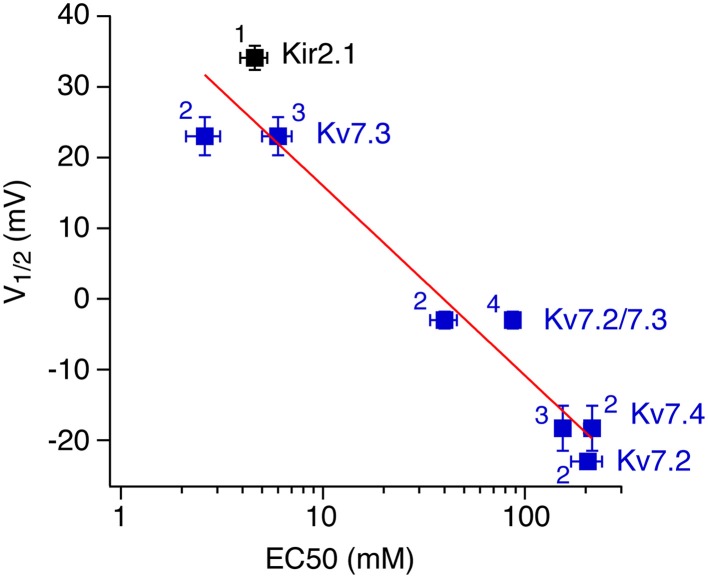
**Correlation between V_1/2_ and apparent affinity for short-chain diC8-PI(4,5)P_2_**. For each of the channels examined, voltage of half-inhibition by Ci-VSP in CHO cells is plotted against half-activating concentrations of diC8-PI(4,5)P_2_ as reported in the literature (1, Lopes et al., 2002; 2, Li et al., 2005; 3, Zhang et al., 2003; Hernandez et al., 2009). V_1/2_-values are taken from Figures 1, 3, respectively. Data for Kv7.4 were obtained as shown for Kv7.2 and Kv7.3 (Figure 3). Red line indicates a fit of a logarithmic function to the data, yielding a slope of −42.8 mV per decade.

For the channels examined as examples here, the rank order of apparent PI(4,5)P_2_ affinities matches well with affinities previously derived by using either chelation of PI(4,5)P_2_ with poly-cations or specific antibodies (Du et al., 2004) or application of the short chain PI(4,5)P_2_ analog, diC8-PI(4,5)P_2_. For some of the channels, previous studies have reported apparent binding affinities for diC8-PI(4,5)P_2_, as partitioning of the short-chain lipid into excised patches is reversible and thus dose-response relations can be obtained (Lopes et al., 2002; Zhang et al., 2003; Li et al., 2005; Hernandez et al., 2008). Plotting our V_1/2_ values for Ci-VSP sensitivity vs. those experimental apparent affinities (Figure 6) shows a steep relation, with about −27 mV shift in V_1/2_ for a 10-fold change of EC_50_ for diC8-PI(4,5)P_2. This calibration allows to directly determine the apparent affinity of a channel or channel mutant from its quantitative response to activation of Ci-VSP. However, it should be emphasized that the affinity for native long-chain PI(4,5)P_2_ may quantitatively be quite different from the affinity for soluble diC8-PI(4,5)P_2_.

Although showing the versatility of this quantitative method, our results also point out important caveats and limitations.

First, because the PI(4,5)P_2_ concentration depends on both degradation and resynthesis activity, there is no general strict relation between membrane potential and PI(4,5)P_2_ level under all experimental conditions, but the PI(4,5)P_2_ levels must also be sensitive to expression density of the exogenous Ci-VSP and to the activity of endogenous kinases and possibly endogenous 5-phosphatase (Halaszovich et al., 2009; Falkenburger et al., 2010). Each of these activities may differ considerably between different cell types and even between individual cells or experiments. However, despite this note of caution our results indicate an acceptable degree of variance between individual experiments or cell batches in the experiments with CHO cells. Also, the obtained V_1/2_ values were surprisingly consistent even between expression systems as different as CHO cells and *Xenopus* oocytes, suggesting that the approach is relatively robust despite the concerns mentioned. Yet it is certainly desirable to standardize VSP expression as much as possible. Because in oocytes, expression levels tend to vary substantially between cell batches even when precisely gauging the amount of injected RNA, we propose that usage of a stable cell line expressing consistent amounts of the VSP may be the most useful way to fix VSP activity between experiments. We note that the response of channels with an affinity close to the resting levels of PI(4,5)P_2_ may be particularly sensitive to variation in basal PI(4,5)P_2_ concentration and expression levels of Ci-VSP. Therefore, it may be difficult to reliably differentiate affinities in this concentration range. However, as exemplified by comparison of Kv7.2 homomeric channels with Kv7.2/3 heteromers, it is possible to reliably distinguish such low-affinity channels from those with an intermediate affinity for PI(4,5)P_2_.

Secondly, the insensitivity of Kir6.2/SUR1 to Ci-VSP activity indicates that the selectivity of the lipid-protein interaction is a main determinant of the channel's sensitivity to VSP activity. Hence, only if the PI effector is selectively regulated by PI(4,5)P_2_, its response to voltage changes is a meaningful readout for PI(4,5)P_2_ affinity. When assessing an effector's dependence on PI(4,5)P_2_ it is therefore advisable to complement the usage of the VSP by testing its responsiveness to recruitment of pseudojanin that depletes both PI(4,5)P_2_ and PI(4)P (Lindner et al., 2011; Hammond et al., 2012). Also, even single point mutations may dramatically change the PI selectivity of channels (Rohacs et al., 2003) and possibly of other PI effectors. Finally it should be kept in mind that VSP also turns over PI(3,4,5)P_3_ to PI(3,4)P_2_, which may also affect the response.

### Distinct PI(4,5)P_2_ pools?

The PI(4,5)P_2_ affinity is thought to be a pivotal determinant of an effector's sensitivity to cellular PI(4,5)P_2_ dynamics. Thus, ion channels with moderate or low affinity for PI(4,5)P_2_, i.e., in the range of the concentration in a resting cell, will readily react to transient depletion of PI(4,5)P_2_, e.g., during activation of Gq-coupled receptors. In contrast, high affinity channels may be quite insensitive to such PI(4,5)P_2_ signals, maintaining their PI(4,5)P_2_ association even when PI(4,5)P_2_ concentration drops to low values (Gamper and Shapiro, 2007). A striking example corroborating this principle has been demonstrated in striatal neurons: here, cholinergic Gq/PLC-mediated modulation of K^+^ currents is prominent in a neuronal population containing Kir2.3 channels that are characterized by low affinity for PI(4,5)P_2_. In contrast, neurons that express Kir2.1 instead, are rather insensitive to cholinergic modulation (Shen et al., 2007).

Accordingly, we expected to find a close and quantitative correlation between PI(4,5)P_2_ affinity as derived from Ci-VSP sensitivity and susceptibility to PI(4,5)P_2_ depletion via PLC. Unexpectedly, our results do not confirm this relationship as a general rule: while sensitivity to Ci-VSP nicely predicted susceptibility to PLC-mediated PI(4,5)P_2_ dynamics within the Kv7 channel subfamily and confirmed previous findings (Hernandez et al., 2009), the behavior of the Kir channels fell outside this pattern. Even if the insensitivity of Kir2.1 may—at least qualitatively—be explained by its high affinity (but see Figure 5C), the R228Q mutant that shifted the affinity into the range of Kv7 family remained insensitive to M1R activation. Hence, another mechanism must contribute to the sensitivity of the channels to PI(4,5)P_2_ dynamics.

**Figure 7:**
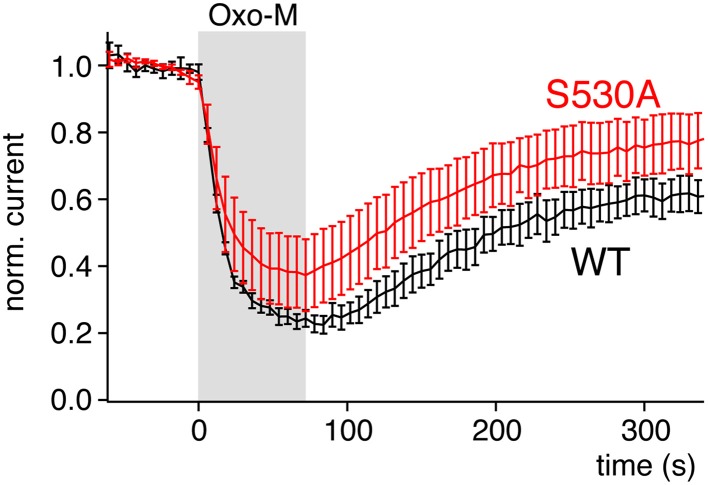
**Mutation of a PKC site of Kv7.2, S530A, does not abolish sensitivity to activation of PLC**. Currents from Kv7.2 (WT) or the PKC site mutant channel Kv7.2(S530A) were recorded from CHO cells co-transfected with M1R. Activation of M1R by 10 μM oxotremorine-M at the time indicated. Currents were evoked by voltage steps from −60 to 0 mV for 500 ms. Currents amplitudes shown were measured at the end of each voltage step and normalized to current amplitude before application of the agonist.

As an obvious explanation that needs to be considered, other biochemical signals accompanying depletion of PI(4,5)P_2_ by the different enzymes may play a role. One possibility is that the sensitivity of Kirs to Ci-VSP is higher than explained by their PI(4,5)P_2_ affinity alone because the PI isoforms produced by VSP activity inhibit these channels. Ci-VSP stoichiometrically generates PI(4)P from PI(4,5)P_2_ as well as PI(3,4)P_2_ from PI(3,4,5)P_3_ (Halaszovich et al., 2009). We therefore need to consider these PIs as inhibitors of Kir2.1. First, we found no evidence for inhibition of Kir2.1 (Figure 4F) or Kir1.1 (data not shown) by PI(4)P. Moreover, combined depletion of PI(4)P and PI(4,5)P_2_ is also highly effective in deactivating Kir2 channels (Kruse et al., 2012), strongly arguing against a role of PI(4)P. Second, the amount of PI(3,4)P_2_ produced by Ci-VSP is very low compared to PI(4,5)P_2_ and PI(4)P (Balla, 2013). Also, application of PI(3,4)P_2_ to patches did not inhibit Kir2.1 (data not shown) and again, PI(4,5)P_2_ depletion without changes in 3-phosphorylated PIs was effective in inhibiting Kir2.1 (Figure 5G). Together the data indicate that the sensitivity of Kir2 channels to Ci-VSP exclusively reflects the channels' sensitivity to PI(4,5)P_2_. Vice versa, we consider that the high responsiveness of Kv7 channels to M1R activation may result from signals in addition to PLC-mediated PI(4,5)P_2_ depletion. In particular, it has been suggested that PKC activation downstream of PLC contributes to channel inhibition by dynamically lowering PI(4,5)P_2_ affinity through phosphorylation of the C-terminus of Kv7.2 (Kosenko et al., 2012). Yet, even if the PKC signal is excluded by elimination of the relevant phosphorylation site or by blocking PKC, activation of M1 receptors still robustly inhibits Kv7 channels (Shapiro et al., 2000; Suh and Hille, 2002; Kosenko et al., 2012). Similarly, we find strong inhibition of Kv7.2 by M1R in a channel mutant lacking the proposed PKC site (Figure 7), which clearly differs from the Kir channel variants with lowered affinity according to sensitivity to Ci-VSP.

Taken together, these considerations suggest another mechanistic explanation for the distinct sensitivities to PLC-mediated depletion of PI(4,5)P_2_. We hypothesize that Kv7 and Kir channels may be modulated by distinct pools of PI(4,5)P_2_ within the plasma membrane. In this model, both pools would be accessible to the VSP as well as to PI(4,5)P_2_ -phosphatases dynamically recruited to the membrane by Lyn-FRB as a membrane anchor. However, the PI(4,5)P_2_ pool associated with Kv7 is predicted to be exclusively accessible to PLC, while the pools associated with Kir channels cannot be depleted by PLC. Structurally, the most obvious model is that both PI(4,5)P_2_ pools are located to spatially separated membrane microdomains with limited diffusional exchange of phosphoinositides (Hilgemann, 2007). There is now a wealth of experimental data that demonstrate local domains enriched in specific PI isoforms (e.g., Golub and Caroni, 2005; Fairn et al., 2009; van den Bogaart et al., 2011; Khuong et al., 2013).

However, the hypothetical distinct pools or domains associated with the ion channels examined here remain to be demonstrated and characterized by more direct approaches. We note there is evidence to suggest that preferential localization of both muscarinic receptors and Kv7 channels in detergent-resistant membrane fractions, classically referred to as “raft” domains, is critical for inhibition of the channels (Oldfield et al., 2009).

## Author contributions

AR, ML, VT, and CH designed and performed experiments and analyzed data. DO and AR conceived the study and DO wrote the manuscript.

### Conflict of interest statement

The authors declare that the research was conducted in the absence of any commercial or financial relationships that could be construed as a potential conflict of interest.

## References

[B1] Arendt K. L. Royo M. Fernandez-Monreal M. Knafo S. Petrok C. N. Martens J. R.. (2009). PIP3 controls synaptic function by maintaining AMPA receptor clustering at the postsynaptic membrane. Nat. Neurosci. 13, 36–44. 10.1038/nn.2462 20010819 PMC2810846

[B2] Balla T. (2013). Phosphoinositides: tiny lipids with giant impact on cell regulation. Physiol. Rev. 93, 1019–1137. 10.1152/physrev.00028.2012 23899561 PMC3962547

[B3] Balla T. Szentpetery Z. Kim Y. J. (2009). Phosphoinositide signaling: new tools and insights. Physiology (Bethesda) 24, 231–244. 10.1152/physiol.00014.2009 19675354 PMC3126675

[B4] Baukrowitz T. Schulte U. Oliver D. Herlitze S. Krauter T. Tucker S. J.. (1998). PIP2 and PIP as determinants for ATP inhibition of KATP channels. Science 282, 1141–1144. 10.1126/science.282.5391.1141 9804555

[B5] Broicher T. Wettschureck N. Munsch T. Coulon P. Meuth S. G. Kanyshkova T.. (2008). Muscarinic ACh receptor-mediated control of thalamic activity via G(q)/G (11)-family G-proteins. Pflugers Arch. 456, 1049–1060. 10.1007/s00424-008-0473-x 18350314

[B6] Brown D. A. Adams P. R. (1980). Muscarinic suppression of a novel voltage-sensitive K+ current in a vertebrate neurone. Nature 283, 673–676. 10.1038/283673a0 6965523

[B7] Di Paolo G. De Camilli P. (2006). Phosphoinositides in cell regulation and membrane dynamics. Nature 443, 651–657. 10.1038/nature05185 17035995

[B8] Du X. Zhang H. Lopes C. Mirshahi T. Rohacs T. Logothetis D. E. (2004). Characteristic interactions with phosphatidylinositol 4,5-bisphosphate determine regulation of Kir channels by diverse modulators. J. Biol. Chem. 279, 37271–37281. 10.1074/jbc.M403413200 15155739

[B9] Fairn G. D. Ogata K. Botelho R. J. Stahl P. D. Anderson R. A. De Camilli P.. (2009). An electrostatic switch displaces phosphatidylinositol phosphate kinases from the membrane during phagocytosis. J. Cell Biol. 187, 701–714. 10.1083/jcb.200909025 19951917 PMC2806594

[B10] Falkenburger B. H. Jensen J. B. Hille B. (2010). Kinetics of PIP2 metabolism and KCNQ2/3 channel regulation studied with a voltage-sensitive phosphatase in living cells. J. Gen. Physiol. 135, 99–114. 10.1085/jgp.200910345 20100891 PMC2812502

[B11] Fan Z. Makielski J. C. (1997). Anionic phospholipids activate ATP-sensitive potassium channels. J. Biol. Chem. 272, 5388–5395. 10.1074/jbc.272.9.5388 9038137

[B12] Gamper N. Shapiro M. S. (2007). Target-specific PIP2 signalling: how might it work? J. Physiol. 582, 967–975. 10.1113/jphysiol.2007.132787 17412762 PMC2075238

[B13] Golub T. Caroni P. (2005). PI(4,5)P2-dependent microdomain assemblies capture microtubules to promote and control leading edge motility. J. Cell Biol. 169, 151–165. 10.1083/jcb.200407058 15809307 PMC2171909

[B14] Halaszovich C. R. Schreiber D. N. Oliver D. (2009). Ci-VSP is a depolarization-activated phosphatidylinositol-4,5-bisphosphate and phosphatidylinositol-3,4,5-trisphosphate 5′-phosphatase. J. Biol. Chem. 284, 2106–2113. 10.1074/jbc.M803543200 19047057

[B15] Hammond G. R. Fischer M. J. Anderson K. E. Holdich J. Koteci A. Balla T.. (2012). PI4P and PI(4,5)P2 are essential but independent lipid determinants of membrane identity. Science 337, 727–730. 10.1126/science.1222483 22722250 PMC3646512

[B16] Hansen S. B. Tao X. MacKinnon R. (2011). Structural basis of PIP2 activation of the classical inward rectifier K+ channel Kir2.2. Nature 477, 495–498. 10.1038/nature10370 21874019 PMC3324908

[B17] Hernandez C. C. Falkenburger B. Shapiro M. S. (2009). Affinity for phosphatidylinositol 4,5-bisphosphate determines muscarinic agonist sensitivity of Kv7 K^+^ channels. J. Gen. Physiol. 134, 437–448. 10.1085/jgp.200910313 19858360 PMC2768799

[B18] Hernandez C. C. Zaika O. Shapiro M. S. (2008). A carboxy-terminal inter-helix linker as the site of phosphatidylinositol 4,5-bisphosphate action on Kv7 (M-type) K+ channels. J. Gen. Physiol. 132, 361–381. 10.1085/jgp.200810007 18725531 PMC2518730

[B19] Hilgemann D. W. (2007). Local PIP(2) signals: when, where, and how? Pflugers Arch. 455, 55–67. 10.1007/s00424-007-0280-9 17534652

[B20] Hilgemann D. W. Ball R. (1996). Regulation of cardiac Na^+^, Ca^2+^ exchange and KATP potassium channels by PIP2. Science 273, 956–959. 10.1126/science.273.5277.956 8688080

[B21] Hobiger K. Utesch T. Mroginski M. A. Friedrich T. (2012). Coupling of Ci-VSP modules requires a combination of structure and electrostatics within the linker. Biophys. J. 102, 1313–1322. 10.1016/j.bpj.2012.02.027 22455914 PMC3309284

[B22] Horowitz L. F. Hirdes W. Suh B.-C. Hilgemann D. W. Mackie K. Hille B. (2005). Phospholipase C in living cells: activation, inhibition, Ca^2+^ requirement, and regulation of M current. J. Gen. Physiol. 126, 243–262. 10.1085/jgp.200509309 16129772 PMC2266577

[B23] Hossain M. I. Iwasaki H. Okochi Y. Chahine M. Higashijima S. Nagayama K.. (2008). Enzyme domain affects the movement of the voltage sensor in Ascidian and zebrafish voltage-sensing phosphatases. J. Biol. Chem. 283, 18248–18259. 10.1074/jbc.M706184200 18375390

[B24] Idevall-Hagren O. Dickson E. J. Hille B. Toomre D. K. De Camilli P. (2012). Optogenetic control of phosphoinositide metabolism. Proc. Natl. Acad. Sci. U.S.A. 109, E2316–E2323. 10.1073/pnas.1211305109 22847441 PMC3435206

[B25] Imai Y. Itsuki K. Okamura Y. Inoue R. Mori M. X. (2012). A self-limiting regulation of vasoconstrictor-activated TRPC3/C6/C7 channels coupled to PI(4,5)P(2)-diacylglycerol signalling. J. Physiol. 590, 1101–1119. 10.1113/jphysiol.2011.221358 22183723 PMC3381819

[B26] Khuong T. M. Habets R. L. Kuenen S. Witkowska A. Kasprowicz J. Swerts J.. (2013). Synaptic PI(3,4,5)P3 is required for Syntaxin1A clustering and neurotransmitter release. Neuron 77, 1097–1108. 10.1016/j.neuron.2013.01.025 23522045

[B27] Kohout S. C. Bell S. C. Liu L. Xu Q. Minor D. L. Jr. Isacoff E. Y. (2010). Electrochemical coupling in the voltage-dependent phosphatase Ci-VSP. Nat. Chem. Biol. 6, 369–375. 10.1038/nchembio.349 20364128 PMC2857593

[B28] Kohout S. C. Ulbrich M. H. Bell S. C. Isacoff E. Y. (2008). Subunit organization and functional transitions in Ci-VSP. Nat. Struct. Mol. Biol. 15, 106–108. 10.1038/nsmb1320 18084307

[B29] Kosenko A. Kang S. Smith I. M. Greene D. L. Langeberg L. K. Scott J. D.. (2012). Coordinated signal integration at the M-type potassium channel upon muscarinic stimulation. EMBO J. 31, 3147–3156. 10.1038/emboj.2012.156 22643219 PMC3400014

[B30] Kruse M. Hammond G. R. Hille B. (2012). Regulation of voltage-gated potassium channels by PI(4,5)P2. J. Gen. Physiol. 140, 189–205. 10.1085/jgp.201210806 22851677 PMC3409096

[B31] Li Y. Gamper N. Hilgemann D. W. Shapiro M. S. (2005). Regulation of Kv7 (KCNQ) K+ channel open probability by phosphatidylinositol 4,5-bisphosphate. J. Neurosci. 25, 9825–9835. 10.1523/JNEUROSCI.2597-05.2005 16251430 PMC6725574

[B32] Lindner M. Leitner M. G. Halaszovich C. R. Hammond G. R. Oliver D. (2011). Probing the regulation of TASK potassium channels by PI(4,5)P2 with switchable phosphoinositide phosphatases. J. Physiol. 589, 3149–3162. 10.1113/jphysiol.2011.208983 21540350 PMC3145931

[B33] Logothetis D. E. Jin T. Lupyan D. Rosenhouse-Dantsker A. (2007). Phosphoinositide-mediated gating of inwardly rectifying K(+) channels. Pflugers Arch. 455, 83–95. 10.1007/s00424-007-0276-5 17520276

[B34] Lopes C. M. B. Zhang H. Rohacs T. Jin T. Yang J. Logothetis D. E. (2002). Alterations in conserved kir channel-PIP2 interactions underlie channelopathies. Neuron 34, 933–944. 10.1016/S0896-6273(02)00725-0 12086641

[B35] Marrion N. V. Smart T. G. Marsh S. J. Brown D. A. (1989). Muscarinic suppression of the M-current in the rat sympathetic ganglion is mediated by receptors of the M1-subtype. Br. J. Pharmacol. 98, 557–573. 10.1111/j.1476-5381.1989.tb12630.x 2819334 PMC1854721

[B36] Mavrantoni A. Thallmair V. Leitner M. G. Schreiber D. N. Oliver D. Halaszovich C. R. (2015). A method to control phosphoinositides and to analyze PTEN function in living cells using voltage sensitive phosphatases. Front. Pharmacol. 6:68. 10.3389/fphar.2015.00068 25873899 PMC4379879

[B37] Murata Y. Iwasaki H. Sasaki M. Inaba K. Okamura Y. (2005). Phosphoinositide phosphatase activity coupled to an intrinsic voltage sensor. Nature 435, 1239–1243. 10.1038/nature03650 15902207

[B38] Murata Y. Okamura Y. (2007). Depolarization activates the phosphoinositide phosphatase Ci-VSP, as detected in *Xenopus oocytes* coexpressing sensors of PIP2. J. Physiol. 583, 875–889. 10.1113/jphysiol.2007.134775 17615106 PMC2277204

[B39] Oldfield S. Hancock J. Mason A. Hobson S. A. Wynick D. Kelly E.. (2009). Receptor-mediated suppression of potassium currents requires colocalization within lipid rafts. Mol. Pharmacol. 76, 1279–1289. 10.1124/mol.109.058008 19726551

[B40] Oliver D. Lien C. C. Soom M. Baukrowitz T. Jonas P. Fakler B. (2004). Functional conversion between A-type and delayed rectifier K+ channels by membrane lipids. Science 304, 265–270. 10.1126/science.1094113 15031437

[B41] Rapedius M. Fowler P. W. Shang L. Sansom M. S. Tucker S. J. Baukrowitz T. (2007). H bonding at the helix-bundle crossing controls gating in Kir potassium channels. Neuron 55, 602–614. 10.1016/j.neuron.2007.07.026 17698013 PMC1950231

[B42] Rohacs T. Lopes C. M. B. Jin T. Ramdya P. P. Molnar Z. Logothetis D. E. (2003). Specificity of activation by phosphoinositides determines lipid regulation of Kir channels. Proc. Natl. Acad. Sci. U.S.A. 100, 745–750. 10.1073/pnas.0236364100 12525701 PMC141067

[B43] Sakata S. Hossain M. I. Okamura Y. (2011). Coupling of the phosphatase activity of Ci-VSP to its voltage sensor activity over the entire range of voltage sensitivity. J. Physiol. 589, 2687–2705. 10.1113/jphysiol.2011.208165 21486809 PMC3112548

[B44] Shapiro M. S. Roche J. P. Kaftan E. J. Cruzblanca H. Mackie K. Hille B. (2000). Reconstitution of muscarinic modulation of the KCNQ2/KCNQ3 K(+) channels that underlie the neuronal M current. J. Neurosci. 20, 1710–1721. Available online at: http://www.jneurosci.org/content/20/5/1710.abstract 10684873 10.1523/JNEUROSCI.20-05-01710.2000 PMC6772928

[B45] Shen W. Tian X. Day M. Ulrich S. Tkatch T. Nathanson N. M.. (2007). Cholinergic modulation of Kir2 channels selectively elevates dendritic excitability in striatopallidal neurons. Nat. Neurosci. 10, 1458–1466. 10.1038/nn1972 17906621

[B46] Shyng S. L. Nichols C. G. (1998). Membrane phospholipid control of nucleotide sensitivity of KATP channels. Science 282, 1138–1141. 10.1126/science.282.5391.1138 9804554

[B47] Stauffer T. P. Ahn S. Meyer T. (1998). Receptor-induced transient reduction in plasma membrane PtdIns(4,5)P2 concentration monitored in living cells. Curr. Biol. 8, 343–346. 10.1016/S0960-9822(98)70135-6 9512420

[B48] Suh B. C. Hille B. (2002). Recovery from muscarinic modulation of M current channels requires phosphatidylinositol 4,5-bisphosphate synthesis. Neuron 35, 507–520. 10.1016/S0896-6273(02)00790-0 12165472

[B49] Suh B.-C. Inoue T. Meyer T. Hille B. (2006). Rapid chemically induced changes of PtdIns(4,5)P2 gate KCNQ ion channels. Science 314, 1454–1457. 10.1126/science.1131163 16990515 PMC3579521

[B50] Suh B. C. Leal K. Hille B. (2010). Modulation of high-voltage activated Ca(2+) channels by membrane phosphatidylinositol 4,5-bisphosphate. Neuron 67, 224–238. 10.1016/j.neuron.2010.07.001 20670831 PMC2931829

[B51] Szentpetery Z. Varnai P. Balla T. (2010). Acute manipulation of Golgi phosphoinositides to assess their importance in cellular trafficking and signaling. Proc. Natl. Acad. Sci. U.S.A. 107, 8225–8230. 10.1073/pnas.1000157107 20404150 PMC2889580

[B52] Tucker S. J. Baukrowitz T. (2008). How highly charged anionic lipids bind and regulate ion channels. J. Gen. Physiol. 131, 431–438. 10.1085/jgp.200709936 18411329 PMC2346576

[B53] van den Bogaart G. Meyenberg K. Risselada H. J. Amin H. Willig K. I. Hubrich B. E.. (2011). Membrane protein sequestering by ionic protein-lipid interactions. Nature 479, 552–555. 10.1038/nature10545 22020284 PMC3409895

[B54] Varnai P. Balla T. (2006). Live cell imaging of phosphoinositide dynamics with fluorescent protein domains. Biochim. Biophys. Acta 1761, 957–967. 10.1016/j.bbalip.2006.03.019 16702024

[B55] Varnai P. Thyagarajan B. Rohacs T. Balla T. (2006). Rapidly inducible changes in phosphatidylinositol 4,5-bisphosphate levels influence multiple regulatory functions of the lipid in intact living cells. J. Cell Biol. 175, 377–382. 10.1083/jcb.200607116 17088424 PMC2064515

[B56] Villalba-Galea C. A. Miceli F. Taglialatela M. Bezanilla F. (2009). Coupling between the voltage-sensing and phosphatase domains of Ci-VSP. J. Gen. Physiol. 134, 5–14. 10.1085/jgp.200910215 19564425 PMC2712979

[B57] Wilke B. U. Lindner M. Greifenberg L. Albus A. Kronimus Y. Bünemann M.. (2014). Diacylglycerol mediates regulation of TASK potassium channels by Gq-coupled receptors. Nat. Commun. 5, 5540. 10.1038/ncomms6540 25420509

[B58] Wissmann R. Bildl W. Oliver D. Beyermann M. Kalbitzer H. R. Bentrop D.. (2003). Solution structure and function of the “tandem inactivation domain” of the neuronal A-type potassium channel Kv1.4. J. Biol. Chem. 278, 16142–16150. 10.1074/jbc.M210191200 12590144

[B59] Wymann M. P. Schultz C. (2012). The chemical biology of phosphoinositide 3-kinases. Chembiochem 13, 2022–2035. 10.1002/cbic.201200089 22965647

[B60] Xie J. Sun B. Du J. Yang W. Chen H. C. Overton J. D.. (2011). Phosphatidylinositol 4,5-bisphosphate (PIP(2)) controls magnesium gatekeeper TRPM6 activity. Sci. Rep. 1, 146. 10.1038/srep00146 22180838 PMC3238349

[B61] Yamaguchi S. Kurokawa T. Taira I. Aoki N. Sakata S. Okamura Y.. (2014). Potential role of voltage-sensing phosphatases in regulation of cell structure through the production of PI(3,4)P2. J. Cell. Physiol. 229, 422–433. 10.1002/jcp.24463 24038012

[B62] Yudin Y. Lukacs V. Cao C. Rohacs T. (2011). Decrease in phosphatidylinositol 4,5-bisphosphate levels mediates desensitization of the cold sensor TRPM8 channels. J. Physiol. 589, 6007–6027. 10.1113/jphysiol.2011.220228 22005680 PMC3286682

[B63] Zhang H. Craciun L. C. Mirshahi T. Rohacs T. Lopes C. M. Jin T.. (2003). PIP(2) activates KCNQ channels, and its hydrolysis underlies receptor-mediated inhibition of M currents. Neuron 37, 963–975. 10.1016/S0896-6273(03)00125-9 12670425

[B64] Zhang Q. Zhou P. Chen Z. Li M. Jiang H. Gao Z.. (2013). Dynamic PIP2 interactions with voltage sensor elements contribute to KCNQ2 channel gating. Proc. Natl. Acad. Sci. U.S.A. 110, 20093–20098. 10.1073/pnas.1312483110 24277843 PMC3864334

